# A 500-year tale of co-evolution, adaptation, and virulence: *Helicobacter pylori* in the Americas

**DOI:** 10.1038/s41396-020-00758-0

**Published:** 2020-09-02

**Authors:** Zilia Y. Muñoz-Ramirez, Ben Pascoe, Alfonso Mendez-Tenorio, Evangelos Mourkas, Santiago Sandoval-Motta, Guillermo Perez-Perez, Douglas R. Morgan, Ricardo Leonel Dominguez, Diana Ortiz-Princz, Maria Eugenia Cavazza, Gifone Rocha, Dulcienne M. M. Queiroz, Mariana Catalano, Gerardo Zerbetto De Palma, Cinthia G. Goldman, Alejandro Venegas, Teresa Alarcon, Monica Oleastro, Filipa F. Vale, Karen J. Goodman, Roberto C. Torres, Elvire Berthenet, Matthew D. Hitchings, Martin J. Blaser, Samuel K. Sheppard, Kaisa Thorell, Javier Torres

**Affiliations:** 1 Unidad de Investigacion en Enfermedades Infecciosas, UMAE Pediatria, Instituto Mexicano del Seguro Social, Ciudad de México, Mexico; 2 Laboratorio de Bioinformática y Biotecnología Genómica, Escuela Nacional de Ciencias Biológicas, Unidad Profesional Lázaro Cárdenas, Instituto Politécnico Nacional, 11340 Mexico City, Mexico; 3 Department of Biology and Biochemistry, The Milner Centre for Evolution, University of Bath, Claverton Down, Bath, UK; 4 Instituto Nacional de Medicina Genomica, Ciudad de México, México; 5 Consejo Nacional de Ciencia y Tecnologia, Catedras CONACYT, Ciudad de México, México; 6 New York University Langone Medical Center, New York, NY USA; 7 UAB Division of Gastroenterology and Hepatology, The University of Alabama at Birmingham, Birmingham, UK; 8 Division of Gastroenterology, Hepatology, and Nutrition, Vanderbilt University, Nashville, TN USA; 9 Western Honduras Gastric Cancer Prevention Initiative Hospital de Occidente Santa Rosa de Copan, Santa Rosa de Copan, Honduras; 10 Laboratorio de Microbiología Molecular, Servicio Instituto de Biomedicina MPPS-UCV, Caracas, Venezuela; 11 Faculdade de Medicina da UFMG, Belo Horizonte, Brazil; 12 Facultad de Medicina, Instituto de Microbiología y Parasitología Médica (IMPAM, UBA-CONICET), Universidad de Buenos Aires-Consejo Nacional de Investigaciones Científicas y Técnicas, Santa Rosa de Copan, Honduras; 13 Instituto de Química y Fisicoquímica Biológicas “Prof. Alejandro C. Paladini”, IQUIFIB UBA-CONICET, Santa Rosa de Copan, Honduras; 14 Facultad de Farmacia y Bioquímica, Cátedra de Física, Universidad de Buenos Aires, C1113AAD Buenos Aires, Argentina; 15 National Scientific and Technical Research Council (CONICET), C1425FQB Buenos Aires, Argentina; 16 Laboratorio de Patogénesis Microbiana, Centro de Investigación Biomédica, Universidad Diego Portales, Ejército, 141 Santiago Chile; 17 Department of Microbiology, Hospital Universitario La Princesa, Instituto de Investigación Sanitaria Princesa, Madrid, Spain; 18 Host-Pathogen Interactions Unit, Faculty of Pharmacy, Research Institute for Medicines (iMed-ULisboa), Universidade de Lisboa, Lisboa, Portugal; 19 Division of Gastroenterology, Centre of Excellence for Gastrointestinal Inflammation & Immunity Research, University of Alberta, Edmonton, AB Canada; 20 Swansea University Medical School, Swansea University, Swansea, UK; 21 Center for Advanced Biotechnology and Medicine, Rutgers University, New Brunswick, NJ USA; 22 Department of Infectious Diseases, Institute of Biomedicine, Sahlgrenska Academy, University of Gothenburg, Gothenburg, Sweden; 23 Department of Microbiology, Tumor and Cell Biology, Karolinska Institutet, Stockholm, Sweden

**Keywords:** Population genetics, Microbial genetics

## Abstract

*Helicobacter pylori* is a common component of the human stomach microbiota, possibly dating back to the speciation of *Homo sapiens*. A history of pathogen evolution in allopatry has led to the development of genetically distinct *H. pylori* subpopulations, associated with different human populations, and more recent admixture among *H. pylori* subpopulations can provide information about human migrations. However, little is known about the degree to which some *H. pylori* genes are conserved in the face of admixture, potentially indicating host adaptation, or how virulence genes spread among different populations. We analyzed *H. pylori* genomes from 14 countries in the Americas, strains from the Iberian Peninsula, and public genomes from Europe, Africa, and Asia, to investigate how admixture varies across different regions and gene families. Whole-genome analyses of 723 *H. pylori* strains from around the world showed evidence of frequent admixture in the American strains with a complex mosaic of contributions from *H. pylori* populations originating in the Americas as well as other continents. Despite the complex admixture, distinctive genomic fingerprints were identified for each region, revealing novel American *H. pylori* subpopulations. A pan-genome Fst analysis showed that variation in virulence genes had the strongest fixation in America, compared with non-American populations, and that much of the variation constituted non-synonymous substitutions in functional domains. Network analyses suggest that these virulence genes have followed unique evolutionary paths in the American populations, spreading into different genetic backgrounds, potentially contributing to the high risk of gastric cancer in the region.

## Introduction

The arrival of the conquistadors in the Americas more than 500 years ago was a major event in human evolution. Colonialism connected human European and African newcomers with Indigenous people (we capitalize Indigenous to respect the preference of some Indigenous research partners who contributed samples for this work) living in the Americas in the precolonial era [1]. So began one of the largest natural admixture events among modern human populations, that continues in the mosaic of diversity and multiculturalism observed in the Americas today [2].

Just as human populations adapt, diversify, and recombine, so too have their microbiota [3]. Nowhere is this more evident than among the stomach-dwelling obligate human pathogen *Helicobacter pylori* where the close relationship of host and bacterium underpins a history of co-evolution that is reflected in the ancestry of strains [4]. Unlike many gastrointestinal microorganisms, the mode of transmission of *H. pylori*, thought to occur principally from human-to-human through close personal contact within families and communities [5], may have led to a degree of genetic isolation and the emergence of *H. pylori* subpopulations that are geographically stratified in different human populations [6, 7]. This has allowed the study of recent human migrations by quantifying changes in the patterns of admixture between historically isolated *H. pylori* populations [8, 9].

In the Americas, rapid evolution in the 500 years since European colonization has influenced the differentiation of *H. pylori* subpopulations across countries [10, 11] to the extent that even individual genes can be identified as having, for example, Latin American ancestry, as observed in analyses of whole-genome ancestry [11, 12]. However, while genetic admixture among *H. pylori* is instructive for understanding human migrations, there is a pressing need to understand the spread of certain genes among subpopulations to better understand adaption in relation to virulence. For example, while *H. pylori* can colonize the stomach for decades without causing any symptoms [13], progression to serious clinical diseases, such as gastric cancer, is associated with carriage of certain genotypes of the bacteria [14, 15], in particular those that carry genes linked with virulence [16].

Here we analyze 723 *H. pylori* genomes, including 254 isolates we collected and sequenced as part of this and previous studies [10, 12]. Isolates were collected from 14 geographical sites across America, from Canada to Argentina, where the risk of gastric cancer is high, particularly in South and Central America. We also sequenced strains from Portugal and Spain to analyze the contribution of the most relevant European ancestral source for Latin America: the Iberian Peninsula. This allowed quantification with a previously unprecedented resolution of different ancestral sources of *H. pylori* in the Americas and of recent and ongoing admixture among ancestral non-American and Indigenous American subpopulations. Against this backdrop of frequent admixture and complex patterns of mosaic inheritance in *H. pylori* we investigated variations in admixture across the genome and the differential spread of genes through multiple ancestral backgrounds. In each regional *H. pylori* population, we were able to identify how important virulence genes have followed unique evolutionary paths in the Americas, potentially contributing to the high risk of gastric cancer in the region [17].

## Materials and methods

### Isolate sampling and genome sequencing

We sampled 149 previously undescribed *H. pylori* isolates from 12 countries in America, from Canada in the North to Argentina in the South and from the Iberian Peninsula (Spain and Portugal) (Supplementary Table 1). These isolates were augmented with 105 published [10, 12] genomes from samples isolated from three Latin American countries to give a total of 254 genomes. Study sites contributed samples from urban areas of mixed ancestry as well as Indigenous communities (Supplementary Table 1). Study site technicians isolated *H. pylori* strains from gastric biopsies of patients or research participants in a selective blood agar media under a microaerobic atmosphere and extracted DNA using the DNeasy Mini Kit (Qiagen, Hilden Germany). Purified DNA from clinical *H. pylori* isolates was sent from the study sites and following quality control, libraries were prepared using Illumina^®^ TruSeq^®^ Nano kit (San Diego, CA, USA) aiming for an insert size of 900 bp. Libraries were sequenced on the MiSeq platform using v3 chemistry, 2*300 bp paired end reads generating a coverage of on average 256-fold (22.3 min to 1063.3 max) (Supplementary Table 2).

### Genome assembly, annotation, and archiving

Illumina raw reads were trimmed and filtered using TrimGalore! software v.0.3.7 (http://www.bioinformatics.babraham.ac.uk/projects/trim_galore/) applying the quality cutoff Q30 and only keeping reads longer than 30 bp. Filtered reads were de novo assembled using SPAdes v.3.9.0 [18]. The resulting draft and complete genomes were annotated as described previously using the online automatic pipeline Rast annotation v.2.0 [19]. Summary statistics from the sequencing and assembly were collected using MultiQC v.1.0 [20]. All genomes with accompanying metadata were submitted to NCBI under the BioProject number PRJNA601302 [21]. The 254 genomes we sequenced were analyzed with 469 *H. pylori* genome sequences published by others. The final study dataset comprised 723 genome sequences that were used for subsequent analyses. Of these, 337 isolates came from populations in the Americas and 386 from populations in other continents (Supplementary Table 1). All genomic sequences were submitted to the online automatic pipeline Rast annotation.

### Core and accessory genome characterization

A pan-genome list was constructed from all 723 isolates in this study by automated annotation of all genomes in the dataset using RAST [19]. Duplicate genes were removed if they exceeded a BLAST threshold of 70% nucleotide identity [22]. This reference pan-genome list comprised 2545 unique genes that appeared in at least one of our 723 genomes. Consistent with previous studies and the whole-genome MLST principle [23], the gene complement and allelic variation of each isolate were determined by comparison with the pan-genome list, with gene presence recorded as a BLAST match exceeding 70% sequence identity over 50% of sequence length. Core genes were present in >90% of the genomes and accessory genes were present in at least one isolate. Each gene was aligned individually using Mafft [24] and concatenated into a single multiFASTA alignment file for each isolate.

### Analysis of genome ancestry

Genome-wide haplotype data were prepared by calling SNPs from a core-genome alignment using BEAGLE v.3.3.2 [25]. A total of 498,461 SNPs were identified in 1385 genes. The genome-wide haplotype data was used in FineSTRUCTURE v.0.02 to define isolate populations based on the similarity of the haplotype copying profiles obtained by CHROMOPAINTER v.0.02 [26]. FineSTRUCTURE was run for 100,000 iterations of both the burn-in and Markov chain Monte Carlo (MCMC) method to cluster individuals based on the co-ancestry matrix as described [27]. The results were visualized as a heat map with each cell indicating the proportion of DNA “chunks” a recipient receives from each donor. The population structure was analyzed in more detail using principal component analysis (PCA) with the co-ancestry matrix obtained previously. Multiple PCAs were calculated from 1 to 11 principal components (PCs) using R.

To identify the proportion of ancestry of *H. pylori* isolates from the Americas, we conducted chromosome painting using ChromoPainterV2 [26], designating both the American and non-American populations as donors (723 genomes), and American isolates as recipients (337 genomes). The algorithm identifies important haplotype information from dense data such as SNP data, and efficiently describes shared ancestry within a recombining population. Each individual is painted using all the other individuals as donors and the result is visualized in a bar plot built with R [28]. Finally, we calculated the average and standard deviation of the proportion of ancestry from each population present in each American country, which were visualized in box plots using R.

### Fst and GenomegaMap analyses to identify gene variants fixed in American populations

Local adaptation in American isolates compared to non-American ancestors was evaluated using the Fst test within the R package PopGenome v.2.2.4 [29]. The Fst calculation was performed by comparing all genes of the pan-genome that were present in >50% of the isolates (1649 genes) in American versus non-American isolates. The criteria for data inclusion in the multiple alignments were as follows. First, we selected genes present in >50% of the studied strains, then the sequences that did not constitute >90% of the length of the gene were removed; finally, each position that did not constitute >50% of alignment depth was also removed. The final alignments were then use to identify polymorphisms using SNP-sites. [30]. A list was obtained by ordering the 1649 genes according to the Fst value, and the top 35 genes (Table 1) were analyzed further. The consensus nucleotide and amino acid sequence alignments of these genes from all the American and non-American isolates were calculated using the SeaView v.4 software [31] and compared against the reference strain 26695 [32] to determine synonymous or non-synonymous mutations. From the top 35 genes, we selected virulence genes with available information on the structure of the proteins. In these genes, the structure of the protein was obtained from Protein Data Bank and the Fst values of each position were visualized in the structure with gradient color, using PyMOL software (The PyMOL Molecular Graphics System, v.2.3.2 Schrödinger, LLC), highlighting positions with an Fst value above the upper 99th percentile.

**Table 1:** List of the 35 genes with the highest Fst values fixed in the American populations.

Top	Gene	No. strains with the gene	No. strains (%)	Description	Fst max value	P99	No. positions >P99
1	HP0543	509	70.40	cag pathogenicity island protein cag22 (cagF)	0.5360	0.4507	3
2	HP0175	723	100.00	Putative peptidyl-prolyl cis,trans-isomerase (PpiC)	0.5018	0.2134	3
3	HP0181	723	100.00	CvpA family protein	0.4957	0.4683	3
4	HP0887	713	98.62	Vacuolating cytotoxin autotransporter (vacA)	0.4885	0.4226	13
5	HP0537	511	70.68	cag pathogenicity island protein cag16 (cagM)	0.4509	0.3841	4
6	id7111332	671	92.81	Hypothetical protein	0.4407	0.4399	1
7	HP0538	510	70.54	cag pathogenicity island protein cag17 (cagN)	0.4394	0.3144	4
8	id7531345	486	67.22	Hypothetical protein	0.4360	0.4304	1
9	HP0524	515	71.23	Type IV secretion system protein	0.4311	0.3095	7
10	HP0547	507	70.12	cag pathogenicity island protein cag26 (cagA)	0.4151	0.2604	9
11	HP0539	510	70.54	cag pathogenicity island protein cag18 (cagL)	0.4126	0.2929	3
12	HP0525	514	71.09	Type IV secretion system ATPase	0.4108	0.3719	3
13	HP0544	510	70.54	Type IV secretion/conjugal transfer ATPase	0.4092	0.2609	8
14	HP0130	722	99.86	Hypothetical protein	0.4039	0.2714	4
15	HP0486	723	100.00	Membrane protein	0.4014	0.3615	5
16	id10887	723	100.00	Putative protein	0.3974	0.2771	2
17	HP0528	509	70.40	cag pathogenicity island protein cag8 (cagX)	0.3901	0.3389	4
18	id970544	721	99.72	Hypothetical protein	0.3892	0.3860	1
19	HP1524	723	100.00	Lipoprotein	0.3852	0.1972	2
20	HP0686	709	98.06	Iron(III) dicitrate transport protein (FecA)	0.3816	0.2385	7
21	id71034	624	86.31	Lysine-specific permease	0.3758	0.2994	5
22	HP0706	723	100.00	Membrane protein	0.3705	0.3092	3
23	HP0559	723	100.00	Acyl carrier protein	0.3693	0.3655	1
24	HP0561	723	100.00	3-oxoacyl-ACP reductase	0.3684	0.2786	3
25	HP0523	517	71.51	cag pathogenicity island protein cag4 (cagg)	0.3622	0.2806	2
26	HP0722	691	95.57	Membrane protein	0.3549	0.3023	5
27	HP1012	722	99.86	Zn-dependent protease	0.3517	0.1692	5
28	HP0211	706	97.65	Beta-lactamase HcpA	0.3512	0.1825	4
29	HP0541	510	70.54	cag pathogenicity island protein cag20 (cagH)	0.3501	0.1942	4
30	HP0554	723	100.00	Hypothetical protein	0.3499	0.2827	4
31	HP0530	510	70.54	Type IV secretion system protein (cagV)	0.3484	0.3401	3
32	HP0534	510	70.54	cag pathogenicity island protein cag13 (cagS)	0.3460	0.2943	2
33	HP1055	721	99.72	Membrane protein	0.3438	0.2876	4
34	HP1243	520	71.92	babA	0.3434	0.2758	4
35	HP0522	520	71.92	cag pathogenicity island protein cag3 (cagd)	0.3416	0.2749	6

Genomegamap was used for Bayesian estimation of *d*_*N*_/*d*_*S*_ ratio (also denoted *K*_*A*_/*K*_*S*_ or *ω*) of *vacA*, *babA*, and *cagA* genes. MCMC was run twice for each analysis with 1000,000 iterations and a burn-in of 22,000 iterations. Both runs were compared for convergence at several parameters and merged to obtain distributions; graphs of omega data were produced using R [33]. Differences in positions under positive selection between American and non-American strains were compared using the MCMC output from the previous analyses.

### Network analysis of genes with high Fst values

#### Genetic distance calculation

The sequence reading frame of the selected virulence genes was verified manually and aligned by reverse translation with MUSCLE, using the SeaView v.4 software. The obtained multiple alignments were used to estimate genetic distance with PAUP v.4.0a166, using maximum likelihood criteria [34].

#### Genetic distance normalization

Genetic distances between each strain pair were obtained from Nexus files and formatted as a triangular matrix. Each distance was normalized between 0 and 1 according to

$$NW_{ij} = 1 - \frac{{W_{ij} - W_{ij(\min)}}} {{W_{ij(\max)}} - W_{ij(\min)}},$$

where *NW*_*ij*_ is the normalized weights between strain *i* and strain *j*, *W*_*ij*_ is the genetic distance between strain *i* and strain *j*, and *W*_*ij*(max)_, *W*_*ij*(min)_ are the maximum and minimum value, respectively, for the genetic distance in the whole network, respectively. With this normalization a value of 0 means the two strains have the highest genetic similarity of all strain pairs, and a value of 1 means the two strains are the most dissimilar.

#### Edge and node trimming

Since all strain pairs have a measure of genetic distance, the resulting network is fully connected and without any perceivable structure. To assess the real structure of the network, edges were removed one by one, starting from the highest values of *NW*_*ij*_ (most dissimilar) until reaching a specific number of connected components. This procedure ensures that only the most genetically similar strain pairs are conserved, while keeping the overall structure of the network based on genetic closeness. If a given edge removal breaks the network into more than one subnetwork, the number of connected components is increased by exactly the number of resulting subnetworks. This edge and node removal procedure is repeated until the number of subnetworks reaches a threshold and the separation of nodes is optimized (*θ*). In the trimmed networks the thresholds are core (*θ* = 3), cagPAI (*θ* = 3), cagA (*θ* = 8), babA (*θ* = 1), vacA (*θ* = 3), and ppiC (*θ* = 3). Networks were drawn using the Fruchterman Reingold [35] algorithm implemented in the network visualization program “gephi” [36].

## Results

### A previously undescribed Southwestern European subpopulation

**Fig. 1:**
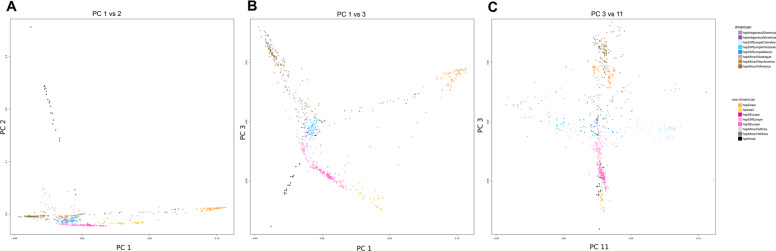
Co-ancestry PCA analysis of 723 *H. pylori* strains from different regions of the world. **a** PC1 vs. PC2, showing the most distant populations hspEAsia and hspAfrica2; **b** PC1 vs. PC3 with the axis showing the separation of continental European and African subpopulations and the distribution of Indigenous American subpopulations; **c** PC3 vs. PC11 with the axis illustrating separation of European and African subpopulations in the Americas. The color code for each population is indicated to the right of the graphic.

We performed co-ancestry analyses of 723 *H. pylori* genomes from different regions of the world to better understand the ancestry of *H. pylori* in Latin America with respect to ancestors from other continents. Sequenced genomes were from countries poorly represented in previous studies, including Spain and Portugal. In the dataset we also included a large number of French isolates, allowing the identification of a new European subpopulation (Fig. 1 and Supplementary Fig. 1). Isolates from the Iberian Peninsula in particular formed the previously undescribed subpopulation termed hspSWEurope, distinct from the subpopulation previously described as hspSEurope [10], which includes isolates from Belgium, Germany, Italy, France, and a few from South Asia. These were also distinct from a third European subpopulation, hspNEurope, which includes mostly isolates from Sweden, the UK, and Ireland.

### Novel American subpopulations

The addition of 254 isolates and genomes from 14 geographical locations (eight not previously represented in whole-genome analyses) greatly improved the resolution of *H. pylori* populations in the Americas (Fig. 1 and Supplementary Fig. 1). This permitted the identification of novel subpopulations across the continent and an improved definition of the ancestral contribution in each regional subpopulation. Three subpopulations with European ancestry were identified, all with a hspSWEurope predominance: hspSWEuropeColombia (previously reported as hspSEuropeColombia) and two novel subpopulations, the hspSWEuropeHonduras including isolates from Honduras and neighboring countries (Nicaragua, Guatemala, and El Salvador) and hspSWEuropeMexico with isolates from Mexico, but also from North, Central, and South America. The greater depth of sampling also allowed improved resolution of the hspAfrica1NAmerica subpopulation, previously described to be present in the US and Canada. This subpopulation was found to be of predominantly West African ancestry (Supplementary Fig. 1), and to include several newly sequenced isolates from Brazil and a few from Colombia. Two other Latin American subpopulations with African, specifically hspAfrica1SAfrica, ancestry were identified: the previously described hspAfrica1Nicaragua population [10, 11] (Supplementary Fig. 1) that includes isolates predominantly from Nicaragua and neighboring Honduras; and the previously reported hspAfrica1MiscAmerica [11], comprising isolates from Mexico and Colombia (Supplementary Fig. 1). These refined descriptions of subpopulations demonstrate ongoing admixture in this region.

Population structure was further studied using PCA analysis (Fig. 1), where the first PC reflects the divergence of Asian strains with hspEAsia at one extreme and African strains at the other. The second PC, on the other hand, documents the distance of the hpAfrica2 population from all others (Fig. 1a). We also observed a component that clearly shows the separation of African and European subpopulations (Fig. 1b). Whereas hspNEurope is closer to hpAsia2, hspSWEurope is closer to European subpopulations of the Americas. The components best illustrating the relationship and distribution of American and non-American subpopulations are depicted in Fig. 1c. This shows hspSWEuropeMexico to be closer to hspSWEurope, while hspSWEuropeHonduras is closer to hspAfrica1Nicaragua, and hspSWEuropeColombia is clearly separated from the others. Separation of African subpopulations in the Americas is also clear: hspAfricaNAmerica is closer to hspAfrica1Wafrica.

The only American isolates that grouped with the Asian populations HpAsia2 and hspEAsia were Indigenous American isolates (often referred to in the literature as Amerind, a term that may have ambiguous interpretations but is generally used to refer to Indigenous Peoples of the Americas) (Fig. 1b). This supports previous suggestions that the Indigenous American *H. pylori* ancestors were replaced by the conquistador *H. pylori* European strains [37] in American populations following European colonization. Furthermore, two Indigenous subpopulations were distinguished: one including strains from Canada (hspIndigenousNAmerica) and the other with strains from South America (hspIndigenousSAmerica), formerly jointly designated hspAmerind  (Supplementary Fig. 1). It has been suggested that Indigenous strains were outcompeted by European strains [38]. To address this we analyzed strains with >10% Indigenous ancestry (Supplementary Fig. 2). The figure shows strains ranging from 100% Indigenous ancestry in Canada (hspIndigenousNAmerica) and over 80% Indigenous ancestry, particularly in the hspIndigenousSAmerica subpopulation, down to around 10% Indigenous ancestry. These strains display a gradient of admixture, with chunks of European, African, and Asian ancestry consistent with in vivo competition; this pattern suggests that Indigenous strains recombined with European and African strains in mestizo populations and were gradually excluded from these populations.

### Countries characterized by distinctive admixture profiles

**Fig. 2:**
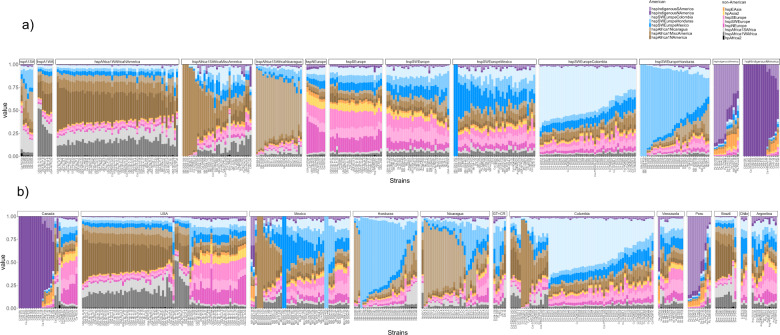
Representation of the ancestry admixture in *H. pylori* strains from American populations, as analyzed with chromosome painting. Each column represents one strain and the color indicates the proportion of the corresponding ancestry in that genome. The color code for each subpopulation is shown on the right of Fig. 2. **a** Representation by subpopulation, from left to right, African, European, and Indigenous American subpopulations; **b** admixture profiles by country along the American continent, from left to right, from Canada to Argentina.

To infer recent recombination in American *H. pylori* subpopulations we used a reference collection of American and non-American donor genomes to paint each American recipient genome, and CHROMOPAINTER v.0.02 to infer the number of DNA chunks donated to each recipient genome from each donor source (Fig. 2a). The three American subpopulations with European ancestry and the three with African ancestry were all painted with a mixture of European and African donors from both American and non-American populations, indicative of a high level of admixture occurring in the Americas. A large proportion of these genomes were painted by donors from their own population, particularly the subpopulations hspSWEuropeColombia, hspSWEuropeHonduras, and hspAfrica1Nicaragua, where 50% or more of the chromosome was painted by their own population (Fig. 2a). This effect was most pronounced in the central parts of the continent, specifically Honduras, Colombia, and Nicaragua (Fig. 2b). The proportion of self-identity (fraction of the genome painted by strains from their own population) is variable in each subpopulation with a gradient from low to high self-identity (Fig. 2a). Admixture was lower in the two Indigenous subpopulations, particularly in the hspIndigenousNAmerica, suggesting these populations have remained genetically isolated, and both carried traces of hspEAsia (Fig. 2b).

**Fig. 3:**
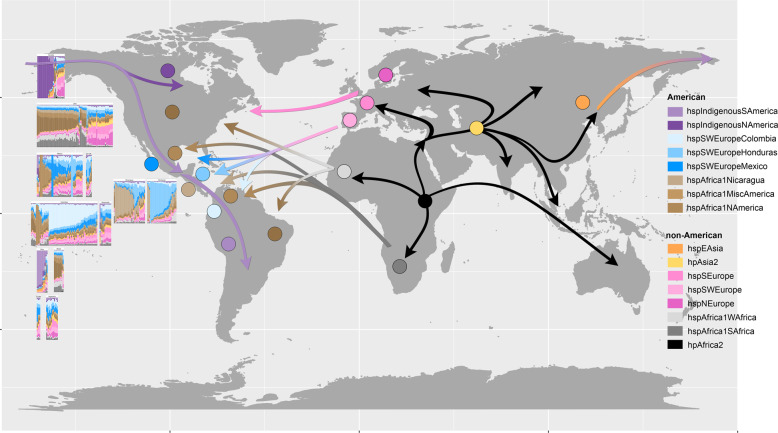
World map illustrating *H. pylori* ancestral contributions to the Americas (colored arrows) from other continents (black arrows). Filled circles represent the dominant *H. pylori* populations at the different geographical locations and the left panel shows chromosome painting results for the isolates from the respective American countries studied.

Even though some isolates in different countries showed similar ancestry, the proportion of inheritance from mixed American and non-American ancestors varied across countries (Fig. 2b and Supplementary Fig. 3), consistent with ongoing admixture between regional subpopulations. Each country can be differentiated from their European and African ancestors as well as American neighbors. Figure 3 and Supplementary Fig. 3 illustrate the composition of genomic ancestry along the continent, showing a particularly high degree of admixture in Mexico—the gateway to the Americas for the Spanish. Figure 3 also summarizes probable migrations to the Americas from other continents.

### Some American isolates retain European or African ancestry

Many American isolates retained strong ancestral roots from other continents and could be classified within European or African populations (Fig. 2a). The first group included isolates from North, Central, and South America, with high levels of hspSWEurope ancestry (Fig. 2b). A second group was classified as hspSEurope, containing mostly isolates from the US, Canada, and Argentina. In addition, a small group of isolates from North America (US and Canada) but none from Latin America was classified as hspNEurope. Finally, a small number of American strains were classified within African subpopulations: some from Honduras as hspAfrica1SAfrica and seven US isolates as hspAfrica1WAfrica (Fig. 2a).

### Virulence genes are fixed in American populations, driving evolution of *H. pylori*

**Fig. 4:**
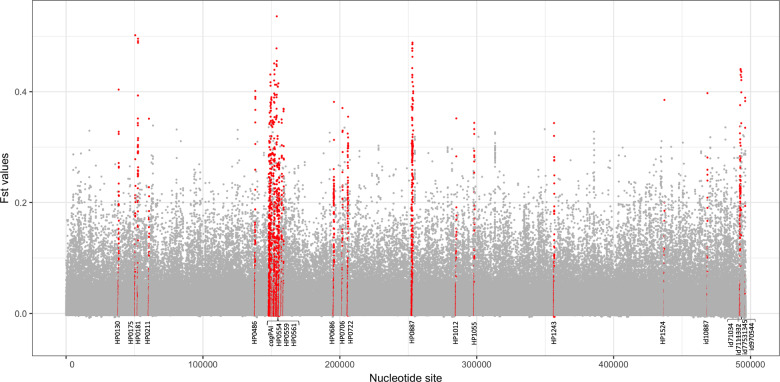
Whole-genome Fst analysis to identify genetic variants that are significantly more common in the Americas than in the rest of the world. The *X*-axis indicates the nucleotide sites and the *Y-*axis shows the Fst value for each site. cagPAI genes include HP0522, HP0523, HP0524, HP0528, HP0525 HP0530, HP0534, HP0537, HP0538, HP0539, HP0541, HP0543, HP0544, and HP0547.

Fst analysis was used to identify genetic variants more common in the Americas than in populations in other continents. Whole-genome analysis (American vs non-American populations) was used with a relaxed threshold for gene presence to include genes present in >50% of the genomes, enabling us to include commonly known virulence genes, such as the *cag*PAI—which are only present in a subset of strains. The distribution of Fst values over the pan-genome (Fig. 4) was used to select 35 genes with the highest Fst values (labeled in red in Fig. 4 and Table 1). In total, there were 142 sites with significant Fst values in these 35 genes, of which 22 encode recognized virulence factors and membrane proteins, including 13 Cag pathogenicity island genes, *vacA*, *babA*, *hofC*, and *ppiC*. Furthermore, the strongest fixation index was identified in the gene encoding for CagF, a chaperone for CagA. Other genes with high numbers of significant sites were *vacA* (with 13 sites), *cagA* (with 9 sites), and the *cag*PAI ATPase (with 8 sites). Fifteen of the 35 genes were present in all strains studied including *vacA*, *hofC*, *ppiC*, three other membrane proteins, *fecA* and a Zn-dependent protease (Table 1). These results suggest that virulence plays a strong role in regional adaption to specific human populations.

### Fixed non-synonymous mutations occur in functionally important domains of virulence proteins

**Fig. 5:**
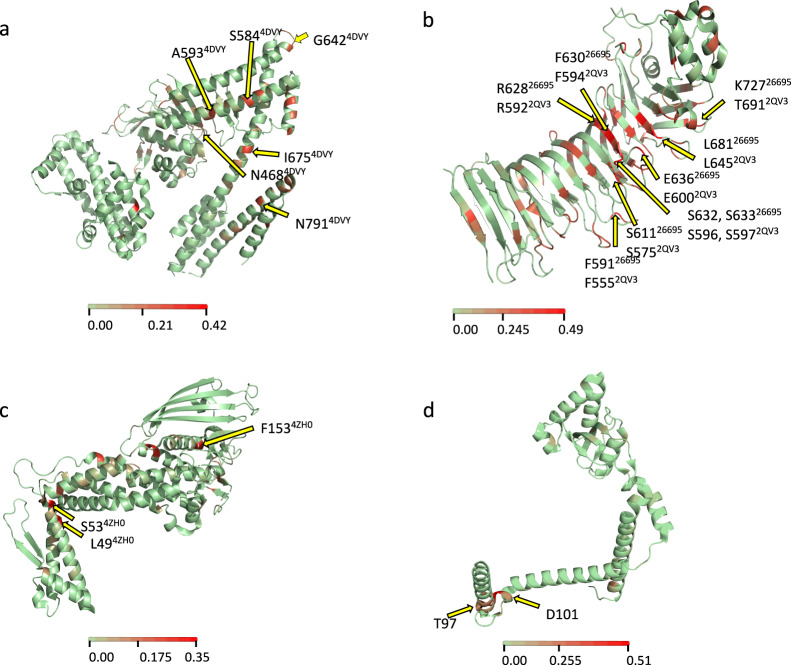
Localization of non-synonymous mutations fixed in the Americas in virulence proteins of *H. pylori*. The available crystal structure of each protein was used to indicate the sites with amino acid changes. **a** CagA, **b** VacA, **c** BabA, and **d** PpiC. The color scale represents the Fst value for each position.

In order to relate mutations significantly fixed in American populations to specific protein domains we further characterized these mutations in virulence genes with corresponding protein structure data. In *cagA* six non-synonymous (NS) substitutions were identified, four in domain II and two in domain III (Fig. 5a and Supplementary Fig. 4a), both regions important for the diverse functions displayed by the protein, specifically the binding of CagA to the inner surface of the host cell and recruitment of PAR1, respectively. In *vacA*, nine of the significant Fst positions led to NS changes and all these amino acid changes were located in the mid region (referred to as the m region) of the protein (Fig. 5b and Supplementary Fig. 4b), the region that targets the protein to the host cell receptor. In BabA, three aa positions—aa 49, 53, and 153—showed a NS substitution (Fig. 5c and Supplementary Fig. 4c); this last residue has been implicated in sensitivity to acid. Finally, in *ppiC*, a peptidyl-prolyl isomerase, three NS substitutions occur in the chaperone domain (two substitutions in D101N and X97A) (Fig. 5d and Supplementary Fig. 4d). The position with the second-highest Fst value among all the genes was a synonymous substitution in aa position 100 of PpiC, also in the chaperone domain (Supplementary Fig. 4d).

To further document specific differences in virulence genes between American and non-American strains, we studied positions under positive selection using GenomegaMap. As with Fst, results showed that major virulence genes (*cagA*, *vacA,* and *babA*) present several positions under strong positive selection (Supplementary Figs. 5–7). Although the regions of the genes with positions under selection were similar for American and non-American strains, the number of positions and strength of the *ω* values were higher for the American strains. This suggests stronger selective pressure in functionally active regions of virulence genes. In *cagA* most of the positions along the gene have as high as 35-fold *ω* values in American isolates, occurring mostly in regions representing extra EPIYA-C and CM motifs (Supplementary Fig. 5) [39]. Similar to Fst analyses, we found positions in the mid region of *vacA* that are under positive selection in the Americas, but not in strains from other continents (Supplementary Fig. 6). In *babA*, GenomegaMap showed positions in the head domain that were under positive selection (Supplementary Fig. 7), consistent with the Fst analyses.

### Host-interacting genes have followed different evolutionary trajectories in *H. pylori* populations

**Fig. 6:**
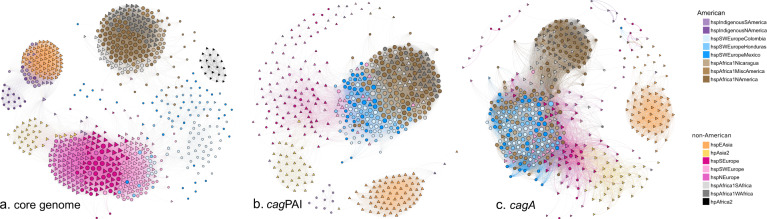
Distance network analyses of the core genome and of virulence genes of the 723 *H. pylori* strains studied. The networks of the core genome, the *cag*PAI pathogenicity island and *cagA* gene are presented. Circles denote American strains and triangles denote strains from other continents. The color of each strain symbol indicates the ancestry as assigned by fineSTRUCTURE and the size of the symbol is proportional to the number of connections each strain has with other strains.

In order to analyze evolution of virulence genes in *H. pylori* populations, a network analysis that compared the distance in gene sequence in an all-versus-all manner to reveal nodes of relatedness was performed. For reference, the analysis was performed with the core genome, resulting in separation of the 14 subpopulations, with the non-American subpopulations grouping in tighter modules relative to the American subpopulations, consistent with a longer evolutionary history with relatively less admixture (Fig. 6a). In contrast, when analyzing cagPAI (Fig. 6b) in the Latin American subpopulations, the three European American subpopulations formed a node in close proximity to another tight node of subpopulations with African ancestry including both American and non-American strains. Whereas continental European populations presented a diffuse group close to hpAsia2 strains, the hspEAsia node was clearly apart from all other subpopulations. When analyzing the *cagA* gene (Fig. 6c), all Latin American subpopulations with African or European ancestry formed a large group, which also included some continental European strains. The American hspAfrica1NAmerica strains clustered with the continental African hspAfrica1WAfrica and hspAfrica1SAfrica strains, and the two Asian nodes clustered separately.

**Fig. 7:**
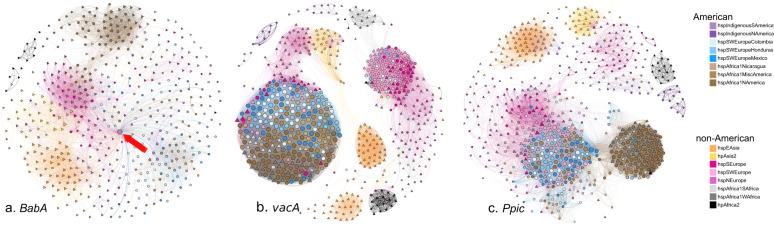
Distance network analyses of virulence genes of the 723 *H. pylori* strains studied. The networks of the *babA*, *vacA*, and *ppiC* genes are presented. Circles denote American strains and triangles denote strains from other continents. The color of each strain symbol indicates the ancestry as assigned by fineSTRUCTURE and the size of the symbol is proportional to the number of connections each strain has with other strains.

When analyzing *babA*, all Latin American subpopulations (with European, African, or Indigenous ancestry) formed a single node closely related to hspEAsia (Fig. 7a). In the Latin American subpopulations, one Indigenous strain showed an unusually large number of interactions with other strains (Fig. 7a, arrow). Conversely, American hspAfrica1NAmerica strains formed a separate module with continental African hspAfrica1WAfrica strains. Furthermore, the continental European strains mixed with hpAsia2 strains, whereas the continental African hpAfrica2 node was clearly distant from all other populations.

When analyzing *vacA* (Fig. 7b), American subpopulations with European or African ancestry clustered in a large node that also contained hspAfrica1WAfrica, hspAfrica1SAfrica, and a few hspSWEurope isolates. In contrast to the other genes, for *vacA*, hspEAsia split into two nodes. Although the continental European populations were dispersed, there were two defined groups, one with mostly hspNEurope strains and the other with hspSEurope, hspSWEurope, and a few Latin American strains. Within analyzing *ppiC* (Fig. 7c), most Latin American subpopulations were within one node that also included some hspSWEurope strains; this group was closer to continental European than to continental African subpopulations. The continental African subpopulations hspAfrica1WAfrica and hspAfrica1SAfrica grouped with the American hspAfrica1NAmerica strains.

In all genes studied, the continental African hpAfrica2 strains formed a group apart from all other populations, probably because this population has remained isolated and without exposure to other populations, thus precluding recombination [40]. Also, in all genes the American hspAfrica1NAmerica strains clustered with the continental African hspAfrica1WAfrica strains, suggesting that in these important host-interacting genes African American strains conserved their original African ancestry.

## Discussion

Genomic variation among *H. pylori* subpopulations in the Americas reflects the ongoing adaption and admixture among strains originating in the Americas and other continents. Although high rates of admixture in human and bacterial populations would be expected to abolish local signals of population structure, distinctive patterns of inheritance and admixture were observed for each country, including small countries that share borders, like Nicaragua and Honduras in Central America. While unequal sampling in some countries may influence the number and geographical specificity of *H. pylori* populations, the clear regional population structure, likely influenced by environmental factors and local diets, is consistent with the history of human migration. The number of *H. pylori* subpopulations per country varied from four in Honduras to six in Mexico and Nicaragua, including Indigenous American (hspIndigenousSAmerica) and continental European (hspSWEurope) subpopulations. This may reflect Mexico’s history as gateway to the Americas and a cross-continental hub of human diversity [41].

The structuring of American *H. pylori* populations with European and African ancestry illustrates a pattern consistent with historic human migrations. For the three Latin American *H. pylori* subpopulations with European ancestry, the major fraction of European ancestry was hspSWEurope, consistent with historical human migrations from Spain and Portugal. For the *H. pylori* subpopulations with African ancestry, two had primarily hspAfrica1SAfrica ancestry while strains from Brazil and the US had primarily hspAfrica1WAfrica ancestry. This pattern is consistent with forced human migration that occurred through the slave trade from the 16th through the 19th century when millions of West Africans were brought to Brazil and the US or the colonies that became the US [42]. Similarly, recent human genomic studies in American populations show that human genomes too reflect a number of migrations, as well as admixture and adaptive processes, leading to modern human Latin American populations [43]. As with *H. pylori*, a Latino-specific European component was recently described in human Caribbean American populations, which significantly diverged from the ancestral Iberian source [44]; thus, the apparent rapid evolution observed in *H. pylori* in America seems to have occurred in humans as well.

In our analysis, some American isolates clustered within continental European and African subpopulations. This observation is consistent with more recent human migrations, such as the movement of Europeans to Canada, the US and some South American countries, including the large European immigration wave to Argentina and Chile during the 19th and 20th centuries [45]. Given the frequency of admixture observed across countries in the Americas, national boundaries cannot explain the maintenance of continental European or African subpopulations. To some degree, the maintenance of these non-American subpopulations likely resulted from reduced admixture of ancestral European or African migrants and their descendants with other American communities. However, it may also be the case that host factors influenced the success of particular *H. pylori* strains and the spread of genes among different subpopulations. Further sampling throughout South America will improve understanding of the importance of human demographic factors in *H. pylori* population structuring.

Concerning the two Indigenous American subpopulations, a broader sampling of *H. pylori* strains from Indigenous American and East Asian communities is needed before drawing conclusions about how they are related to American and non-American subpopulations. It should be noted, in particular, that only one Indigenous community in Canada (Aklavik, Northwest Territories) contributed samples for this work. Interestingly, Okinawan strains seem to constitute an intermediate group between hspEAsia and Indigenous American strains, which can be seen, for example, in Fig. 1a (purple circles).

Pan-genome-wide Fst analysis quantified the degree of differentiation among *H. pylori* genes in different subpopulations revealing variation in the patterns of gene flow (Fig. 3). Most of the genes with the highest fixation values encoded proteins that interact with the host, and in particular, known virulence factors. These included several genes of the cag pathogenicity island [46], which have high affinity interactions with proteins of the human host. Fixed nucleotide variants mainly resulted in non-synonymous mutations in functionally important domains. For example, in CagA, changes occurred in domains II and III. Domain II is known to tether CagA to the inner surface of the host cell by binding to phosphatidylserine [47] and also has a region that recognizes β1-integrin, a binding necessary to allow translocation of CagA. In domain III, one NS substitution (N791D) was in the N-terminal binding sequence (NBS). This may affect the intramolecular interaction NBS/CBS that is reported to be important in the recruitment of PAR1 [39]. PAR1 recruitment tethers two CagA proteins via the CM sequence to enhance CagA-SHP2 complex formation [48], leading to activation of the Ras-ERK MAPK pathway. This domain has also some similarity with cytoskeletal proteins that may participate in the alteration of cell–cell junctions by mimicking host proteins.

In VacA, nucleotide substitutions occurred in the m region of the protein, which recognizes the receptor on the surface of the human cell [49, 50]; changes in this domain may also change specificity to the target cell [50–52]. Interestingly, six of the NS substitutions occurred in the denominated subdomain four in p55 between β-sheets 32–35, a surface-exposed domain, which may be exposed to selection and diversification driven by the immune response [53]. In BabA, three NS mutations caused a change in aa residues 49, 53, and 153. Whereas 49 and 53 have not yet been associated with any function, it has recently been hypothesized that the change F153M causes increased acid sensitivity of Leb binding, altering the pH-responsive mechanism of adherence to the gastric mucosa [54]. Finally, in PpiC, a peptidyl-prolyl isomerase with two functionally important domains, the enzymatic activity and the chaperone function [55, 56], three NS substitutions occurred in the chaperone domain. Thus, most of the fixed base changes occurred in functionally relevant regions of virulence genes, in domains known to interact with molecules of the host. Furthermore, in the major virulence genes *cagA*, *vacA,* and *babA*, American strains presented higher *ω* values at a larger number of positions, indicating a stronger selection in the Americas, consistent with more recent host-bacteria adaption in this region. It is plausible that some of the human genes that encode proteins interacting with *H. pylori* proteins are under a co-evolutive process for a better co-existence of *H. pylori* and its human host. Along these lines, recent studies in American human populations suggest a rapid adaptive evolution in genes involved in inflammation, blood metabolites, and immune related-traits, showing that admixture is a key driving mechanism in this rapid evolution in Latin American human populations [43, 57].

Combined with an understanding of the background population structure, evidence of different evolutionary trajectories among *H. pylori* genes, potentially influenced by host factors, allowed investigation of the spread of virulence genes into different genetic backgrounds. Visualization of interactions between populations using gene-sequence distance network analysis revealed that among Latin American *H. pylori* subpopulations with European ancestry, the cagPAI is evolving to form a separate population, distinct from African and Asian populations. This contrasts with observations of the *cagA* gene in isolation, in which all Latin American subpopulations, regardless of African or European ancestry, grouped in a module including continental European strains. This suggests that the European ancestral variant of this gene may be preferred over the African ancestral variant in Latin American strains, allowing the former to spread more readily into multiple genetic backgrounds.

Patterns of variation at different loci provided information about the levels of conservation and diversification in multiple genetic backgrounds. For example, *babA* from all Latin American subpopulations comprised a single cluster, in which the Indigenous South American strains interact with both hspEAsia strains and with Latin American strains, suggesting that Indigenous South American strains conserve EAsia characteristics but also share ancestry with the contemporary Latin American subpopulations. A different structure was observed for *vacA* for which all American populations, except the Indigenous strains, tightly grouped with hspAfrica1WAfrica and hspAfrica1SAfrica strains, indicating that *vacA* retains strong African ancestry across the Americas, excluding Indigenous communites. In contrast, for *ppiC* there was evidence of the retention of European ancestry, given that all Latin American populations clustered with continental European strains.

Since most non-synonymous substitutions occur in regions important for interaction with the human molecular target, it is likely that selection acts upon both host and pathogen targets, as it does for some other human infections [58, 59]. Thus, since most conserved *cagA* mutations occur in regions interacting with β1-integrin and with PAR1, it is likely that these proteins conserve the continental European interacting motifs in Latin American mestizo populations, regardless of African or European ancestry. Considering *babA*, it is likely that it differentiated in Latin American mestizo populations to interact with the largely predominant type O blood group antigen [60]. In VacA, changes are in the region recognizing the cell receptor; our results suggest that in all mestizo and African American populations the receptor motif is conserved and shares homology with the West African population. Interestingly, *vacA* was the only gene with two clearly separated hspEAsia subpopulations, suggesting that there may be different VacA receptors or motif variants in humans with East Asian ancestry.

Extensive sampling of strains from the Iberian Peninsula and countries across the Americas has provided evidence of frequent admixture between *H. pylori* populations with a complex mosaic of contributions from non-American subpopulations, along with more recent ongoing contributions from American populations. Against this backdrop of population structure, which reflects the history of humans populating the Americas, there is evidence of host-pathogen co-evolution. It is known that infections represent a major selective pressure for humans and that evidence of this is often located in protein domains that interact with pathogens [59]. By identifying the different evolutionary trajectories in *H. pylori* pathogenicity genes and the highly specific evolutionary changes associated with different human ancestral populations, we provide a basis for considering how human migrations can lead to the emergence of novel pathogen subpopulations. Latin America is a region with one of the highest mortality rates due to gastric cancer in the world [17] and understanding the nature of the co-evolution of *H. pylori* virulence genes with interacting proteins in its human host will help us find ways to counteract their deleterious effects.

## Supplementary information

Suppl. Fig. 1. Population structure of H. pylori strains from the Americas and other continents.

Suppl. Fig 2. Representation of the ancestry admixture in H. pylori strains from Indigenous American populations, as analysed with chromosome painting.

Suppl. Fig 3. Comparative admixture analyses of H. pylori strains in the Americas by country.

Suppl. Fig. 4. Fst analysis of individual virulence genes to identify genetic variants that are significantly more commonly in the Americas than in the rest of the world.

Suppl. Fig. 5. Analyses of positions under selective pressure in the cagA gene of strains from the Americas and other continents using GenomegaMap.

Suppl. Fig. 6. Analyses of positions under selective pressure in the vacA gene of strains from the Americas and other continents using GenomegaMap.

Suppl. Fig. 7. Analyses of positions under selective pressure in the babA gene of strains from the Americas and other continents using GenomegaMap.

Suppl. Table 1. Characteristics of the 723 worldwide H. pylori strains analyzed in this study.

Suppl. Table 2. Genome size, contigs number and coverage of the genomes from the 149 H. pylori strains sequenced in this study.
