# Supplementary material for: A 500-year tale of co-evolution, adaptation, and virulence: Helicobacter pylori in the Americas
Source: ISME J. 2020 Sep 2;15(1):78–92. doi: 10.1038/s41396-020-00758-0 (PMC7853065; doi:10.1038/s41396-020-00758-0)
Supplement: Supplementary file 1 — Suppl. Fig. 1. Population structure of H. pylori strains from the Americas and other continents. [file 41396_2020_758_MOESM1_ESM.pdf]

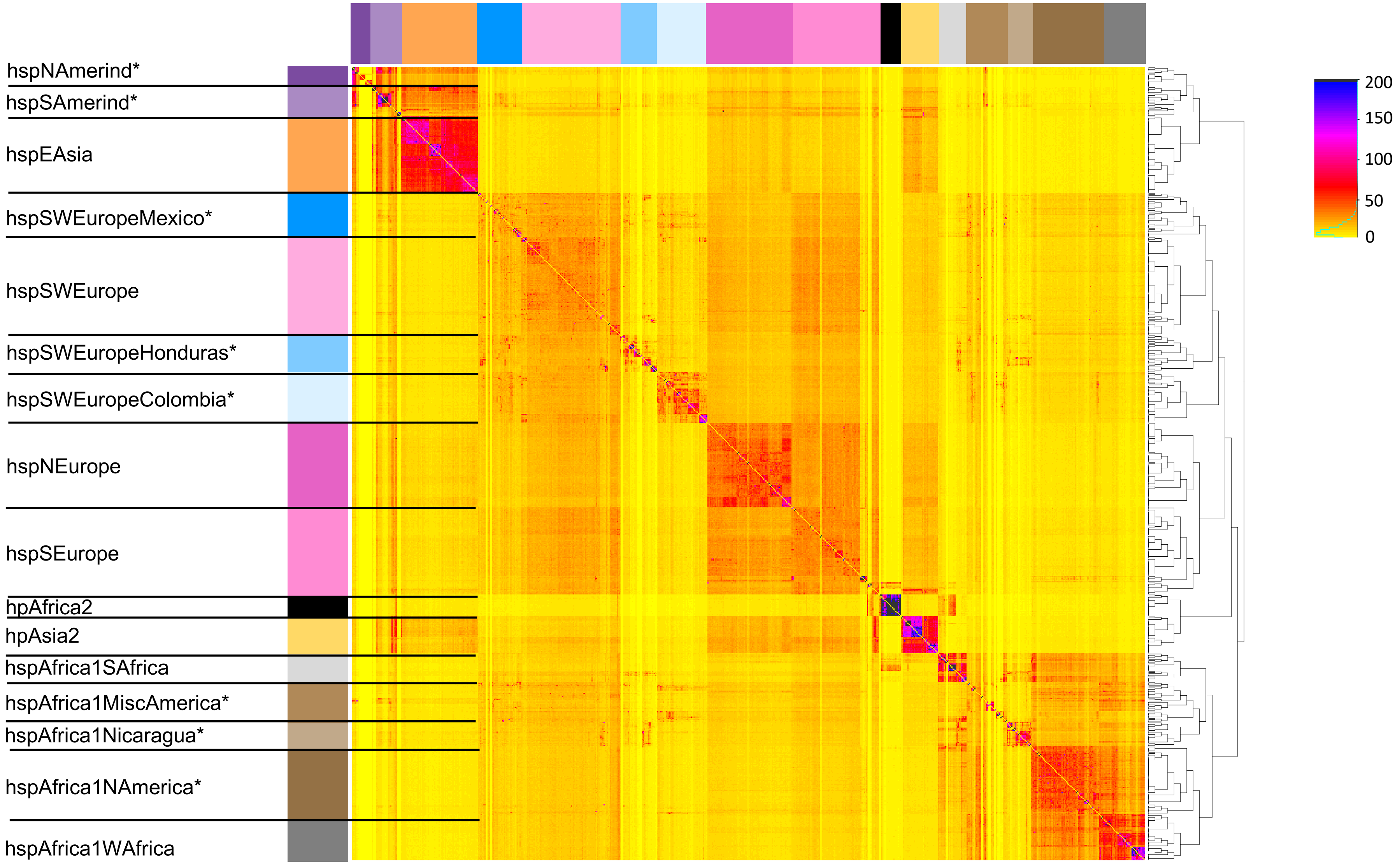

Suppl. Fig. 1. Population structure of *H. pylori* strains from the Americas and other continents. The genomes of the 723 strains were analysed with fineSTRUCTURE software. The colour of each cell of the matrix indicates the number of DNA chunks imported from the corresponding donor genome (column) to the recipient genome (row). The named populations are indicated on the left, with the American subpopulations indicated with an asterisk. The colour bar on the left and top is the assigned colour to each named subpopulation, which is also used in each of the other figures.
