# Supplementary material for: A 500-year tale of co-evolution, adaptation, and virulence: Helicobacter pylori in the Americas
Source: ISME J. 2020 Sep 2;15(1):78–92. doi: 10.1038/s41396-020-00758-0 (PMC7853065; doi:10.1038/s41396-020-00758-0)
Supplement: Supplementary file 2 — Suppl. Fig 2. Representation of the ancestry admixture in H. pylori strains from Indigenous American populations, as analysed with chromosome painting. [file 41396_2020_758_MOESM2_ESM.pdf]

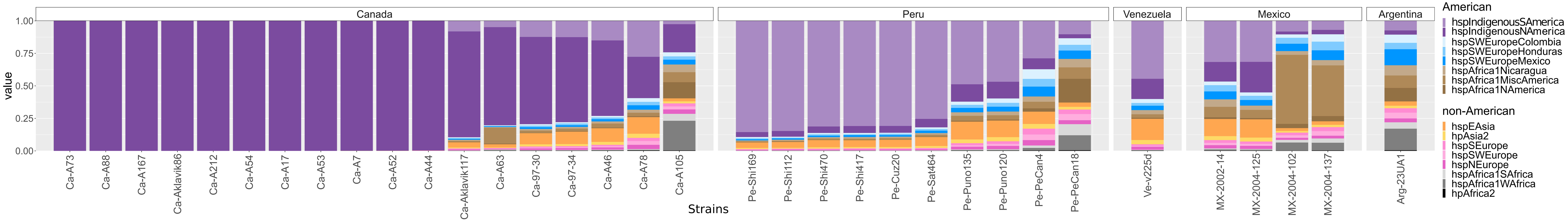

Suppl. Fig 2. Representation of the ancestry admixture in H. pylori strains from Indigenous American populations, as analysed with chromosome painting.
Each column represents one strain and the colour indicates the proportion of the corresponding ancestry in that genome. The colour code for each subpopulation is shown on the right of the figure. Admixture profiles by country of strains with >10% of Indigenous ancestry.
