# Supplementary material for: A 500-year tale of co-evolution, adaptation, and virulence: Helicobacter pylori in the Americas
Source: ISME J. 2020 Sep 2;15(1):78–92. doi: 10.1038/s41396-020-00758-0 (PMC7853065; doi:10.1038/s41396-020-00758-0)
Supplement: Supplementary file 3 — Suppl. Fig 3. Comparative admixture analyses of H. pylori strains in the Americas by country. [file 41396_2020_758_MOESM3_ESM.pdf]

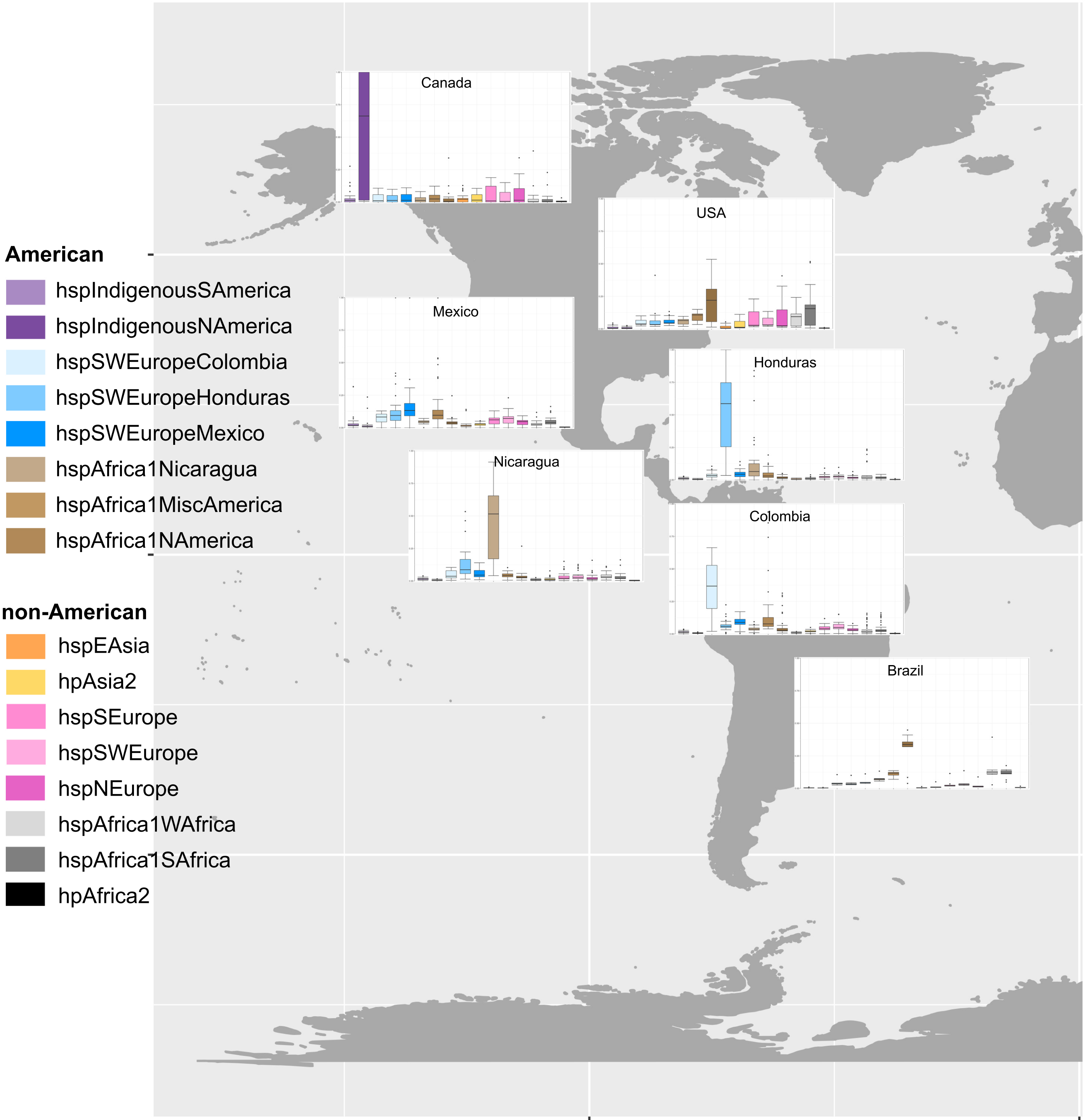

Suppl. Fig 3. Comparative admixture analyses of *H. pylori* strains in the Americas by country.
The proportion of ancestry of each subpopulation present by country was estimated and the mean plus deviation standard is represented in Box plots.
