# Supplementary material for: A 500-year tale of co-evolution, adaptation, and virulence: Helicobacter pylori in the Americas
Source: ISME J. 2020 Sep 2;15(1):78–92. doi: 10.1038/s41396-020-00758-0 (PMC7853065; doi:10.1038/s41396-020-00758-0)
Supplement: Supplementary file 4 — Suppl. Fig. 4. Fst analysis of individual virulence genes to identify genetic variants that are significantly more commonly in the Americas than in the rest of the world. [file 41396_2020_758_MOESM4_ESM.pdf]

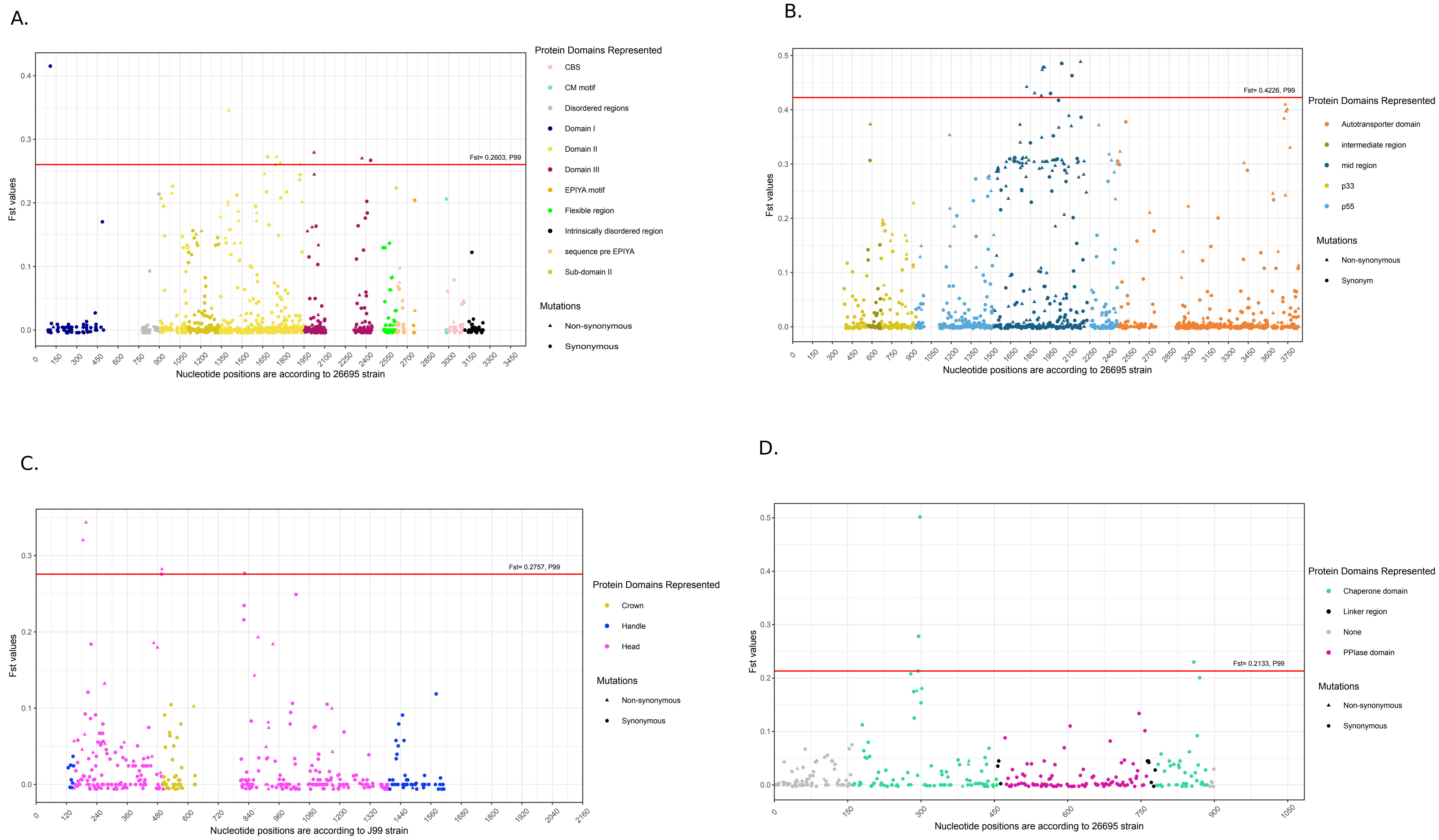


**Suppl. Fig. 4. Fst analysis of individual virulence genes to identify genetic variants that are significantly more commonly in the Americas than in the rest of the world.** The X axis indicates the nucleotide position in the gene, whereas the Y axis shows the Fst value for each position. a) cagA, b) vacA, c) babA, and d) ppiC. Known domains for each protein were taken from: for CagA, [41]; for VacA, [62]; for BabA, [63]; and for PpiC, [64].
