# Supplementary material for: A 500-year tale of co-evolution, adaptation, and virulence: Helicobacter pylori in the Americas
Source: ISME J. 2020 Sep 2;15(1):78–92. doi: 10.1038/s41396-020-00758-0 (PMC7853065; doi:10.1038/s41396-020-00758-0)
Supplement: Supplementary file 5 — Suppl. Fig. 5. Analyses of positions under selective pressure in the cagA gene of strains from the Americas and other continents using GenomegaMap. [file 41396_2020_758_MOESM5_ESM.pdf]

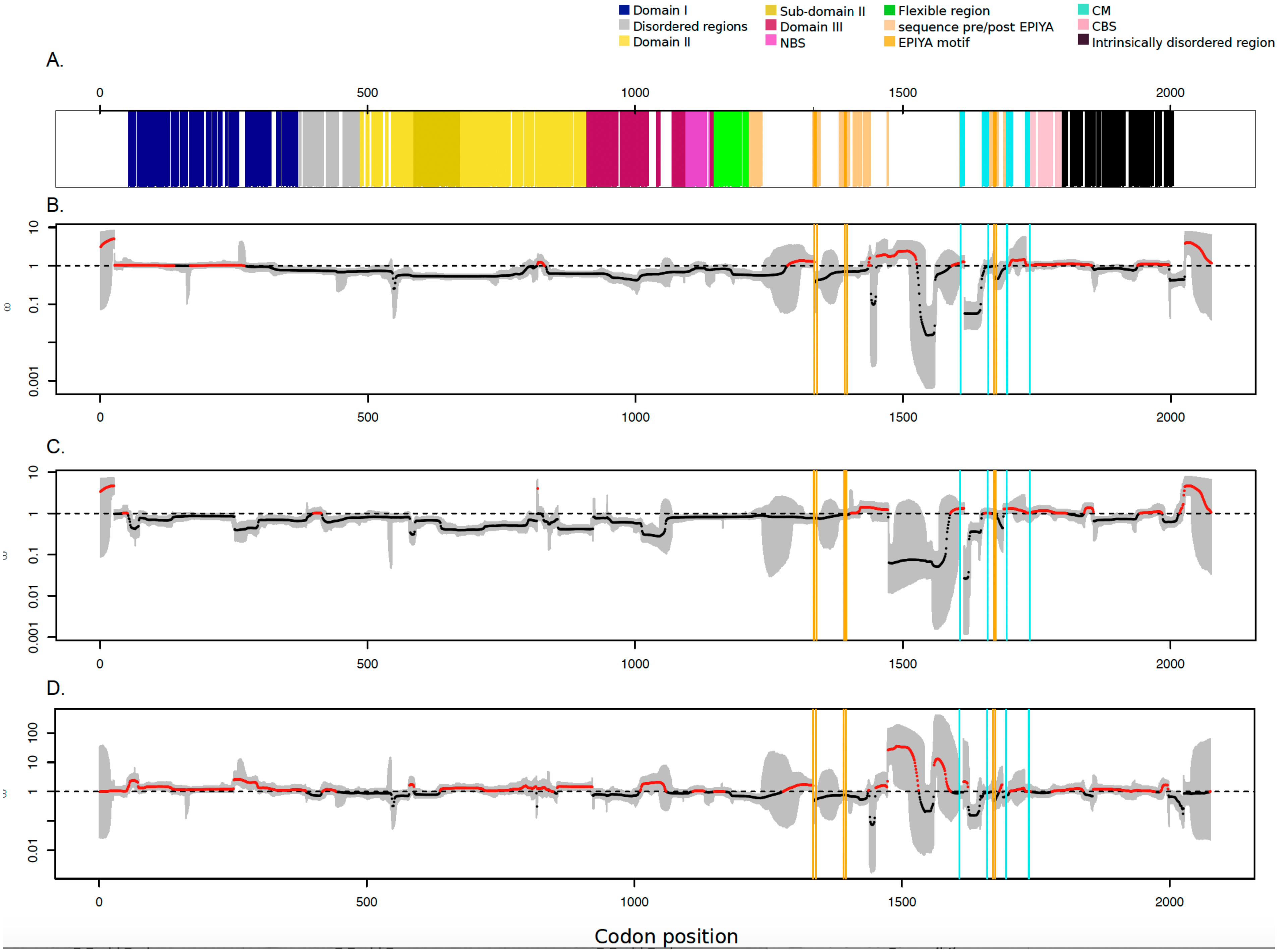

Suppl. Fig. 5. Analyses of positions under selective pressure in the *cagA* gene of strains from the Americas and other continents usingGenomegaMap. a) Known domains of CagA [42]; b) omega values for each position of cagA in strains from the Americas;c) omega values for each position of cagA in non-American strains; d) America/non-America ratio of omega values showing positive pressure ishigher in most regions of the gene in American strains relative to non-American strains. In b, c and d the orange horizontal lines show the 3 EPIYAmotifs and the blue lines the CM motifs, according to the 26695 strain.
