# Supplementary material for: A 500-year tale of co-evolution, adaptation, and virulence: Helicobacter pylori in the Americas
Source: ISME J. 2020 Sep 2;15(1):78–92. doi: 10.1038/s41396-020-00758-0 (PMC7853065; doi:10.1038/s41396-020-00758-0)
Supplement: Supplementary file 6 — Suppl. Fig. 6. Analyses of positions under selective pressure in the vacA gene of strains from the Americas and other continents using GenomegaMap. [file 41396_2020_758_MOESM6_ESM.pdf]

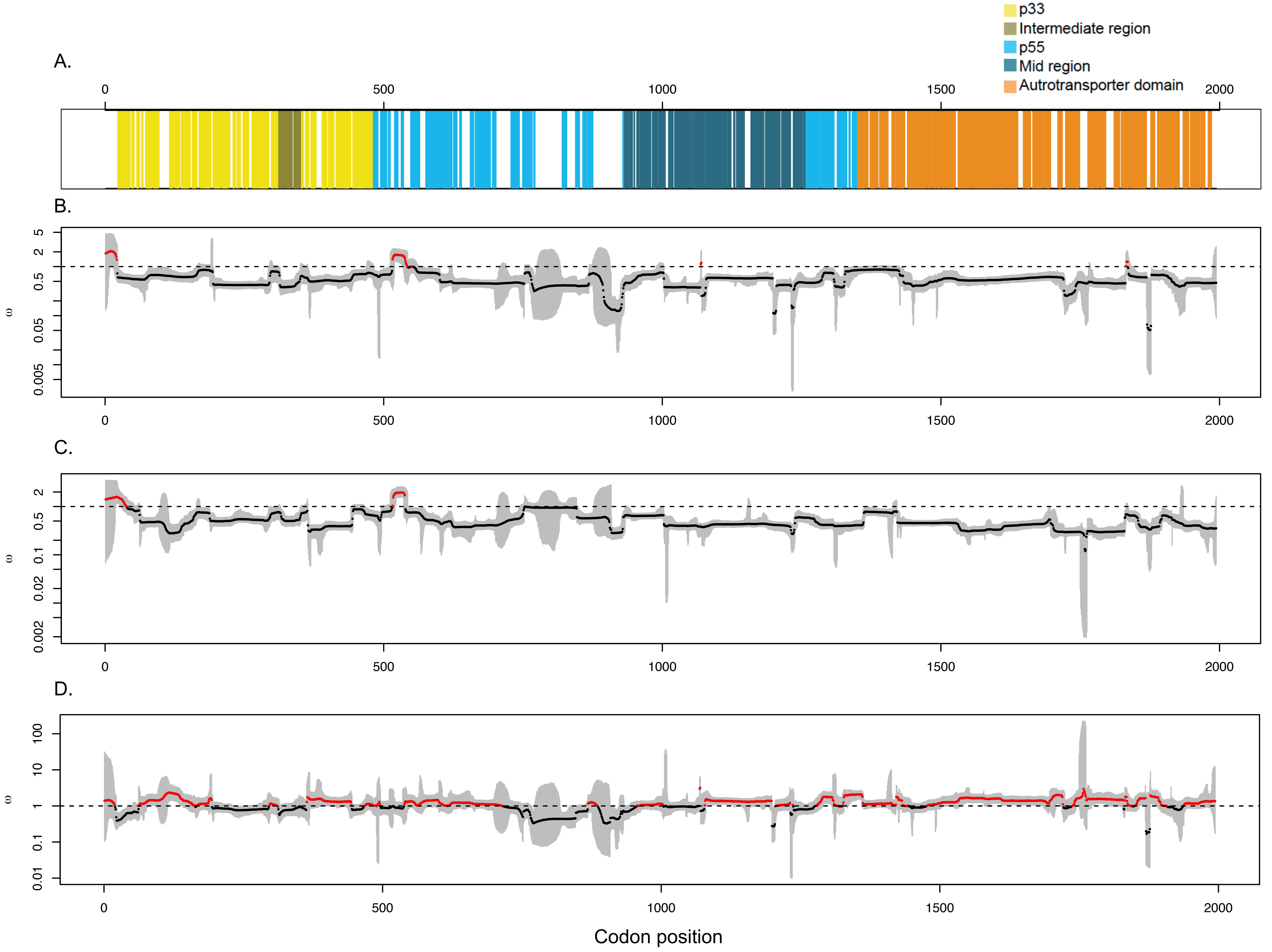

Suppl. Fig. 6. Analyses of positions under selective pressure in the *vacA* gene of strains from the Americas and other continents using GenomegaMap.
a) Known domains of VacA [54]; b) omega values for each position of vacA in strains from the Americas; c) omega values for each position of vacA in strains fromthe other continents; d) America/non-America ratio of omega values showing positive pressure is higher in most regions of the gene in American strains relative to non-American strains.
